# Nardilysin in adipocytes regulates UCP1 expression and body temperature homeostasis

**DOI:** 10.1038/s41598-022-07379-x

**Published:** 2022-03-02

**Authors:** Sayaka Saijo, Mikiko Ohno, Hirotaka Iwasaki, Shintaro Matsuda, Kiyoto Nishi, Yoshinori Hiraoka, Natsuki Ide, Takeshi Kimura, Eiichiro Nishi

**Affiliations:** 1 Department of Cardiovascular Medicine, Graduate School of Medicine, Kyoto University, 54 Shogoin-Kawahara-cho, Sakyo-ku, Kyoto, 606-8507 Japan; 2 Department of Pharmacology, Shiga University of Medical Science, Seta Tsukinowa-Cho, Otsu, Shiga 520-2192 Japan; 3 Division of Clinical Pharmacy, Faculty of Pharmaceutical Sciences, Kobe Gakuin University, 1-1-3 Minatojima, Chuo-ku, Kobe, 650-8586 Japan; 4 Present Address: Japanese Red Cross Otsu Hospital, 1-1-35, Nagara-cho, Otsu, Shiga 520-0000 Japan; 5 Present Address: Division of Endocrinology, UCLA, 650 Charles E. Young Dr. S. CHS 34-115, Los Angeles, CA 90095 USA

**Keywords:** Molecular biology, Physiology

## Abstract

Brown adipose tissue (BAT) dissipates chemical energy as heat through uncoupling protein 1 (UCP1). The induction of mitochondrial reactive oxygen species (ROS) in BAT was recently identified as a mechanism that supports UCP1-dependent thermogenesis. We previously demonstrated that nardilysin (NRDC) plays critical roles in body temperature homeostasis. Global NRDC-deficient (*Nrdc*^–/–^) mice show hypothermia due to a lower set point for body temperature, whereas BAT thermogenesis at room temperature (RT) is enhanced mainly to compensate for poor thermal insulation. To examine the primary role of NRDC in BAT thermogenesis, we generated adipocyte-specific NRDC-deficient (Adipo-KO) mice by mating Nrdc floxed (*Nrdc*^flox/flox^) mice with adiponectin-Cre mice. Adipo-KO mice showed hyperthermia at both RT and thermoneutrality. They were also more cold-tolerant than *Nrdc*^flox/flox^ mice. However, UCP1 mRNA levels were significantly lower in Adipo-KO BAT at RT, thermoneutrality, and 4 °C, whereas no significant differences were observed in UCP1 protein levels at RT and 4 °C. We examined the protein stability of UCP1 using the cycloheximide chase assay and found that NRDC negatively regulated its stability via the ubiquitin–proteasome pathway. NRDC may be also involved in ROS-mediated in vivo thermogenesis because the inhibitory effects of N-acetyl cysteine, an ROS scavenger, on β3 agonist-induced thermogenesis were stronger in Adipo-KO mice. Collectively, the present results demonstrate that NRDC in BAT controls adaptive thermogenesis and body temperature homeostasis possibly via the regulation of UCP1 protein stability and ROS levels.

## Introduction

Core body temperature in mammals is maintained within a narrow range, which is referred to as the set point. Changes in ambient temperature activate skin thermoreceptors, which transmit afferent signals to the thermoregulatory center, the preoptic area of the hypothalamus^1,2^. Efferent signals from the preoptic area then control thermal effector organs, including brown adipose tissue (BAT), skeletal muscle, and skin vessels, to maintain a balance between heat production and dissipation. Under cold stress, mammals prevent heat loss by decreasing skin blood flow and generate excess heat through adaptive (non-shivering) and shivering thermogenesis^3^. BAT is a specific organ for adaptive thermogenesis that dissipates chemical energy as heat through mitochondrial uncoupling protein 1 (UCP1)^3–6^. The expression and activity of UCP1 are regulated at multiple levels. UCP1 mRNA levels are transcriptionally regulated through several transcription factors and co-regulators, such as peroxisome proliferator-activated receptors (PPARs), cAMP response element-binding protein (CREB), and PPAR-γ coactivator-1α (PGC-1α)^7^. The protein stability of UCP1 appears to be regulated by the ubiquitin–proteasome pathway^8^, while its proton transporter activity is negatively and positively controlled by purine nucleotides and free fatty acids, respectively^9^. Recent studies also identified mitochondrial reactive oxygen species (ROS) production and succinate as important upstream regulators of UCP1-dependent thermogenesis^10,11^.

Nardilysin (NRDC; alternative name: N-arginine dibasic convertase, human gene symbol: *NRDC*, mouse gene symbol: *Nrd1*) is a zinc peptidase of the M16 family^12^. We identified NRDC as a specific binding partner of HB-EGF^13^. NRDC is a multifunctional protein that enhances the ectodomain shedding of various membrane proteins in the extracellular space^14–19^, while it controls transcription as a co-regulator in the nucleus^20–23^.

We previously demonstrated that the whole-body deletion of NRDC (*Nrdc*^−/−^) resulted in hypothermia and severe cold intolerance despite enhanced thermogenesis in BAT^21^. This complex phenotype may be attributed to the lower set point for body temperature, poor insulation, and the impaired cold induction of BAT thermogenesis. Based on these findings, we concluded that BAT thermogenesis peaks at room temperature (RT) to compensate for poor thermal insulation, which is the reason for cold intolerance^21^. However, difficulties have been associated with excluding the systemic effects of other organ-derived NRDC on BAT thermogenesis because NRDC is expressed in a wide range of tissues, including the nervous, vascular, and endocrine systems. NRDC in the peripheral nervous system may be involved in thermogenesis because the sympathetic nervous system is activated in *Nrdc*^−/−^ mice^19^.

In the present study, we generated adipocyte-specific NRDC-deficient mice (Adipo-KO) to clarify the primary role of NRDC in BAT thermogenesis. Adipo-KO mice were hyperthermic and more cold-tolerant than control mice. Although UCP1 mRNA levels were significantly lower in Adipo-KO BAT, its protein levels were similar, which may be due to the increased protein stability of UCP1. NRDC may be also involved in ROS-mediated in vivo thermogenesis. Therefore, the present results demonstrate that NRDC in BAT controls adaptive thermogenesis possibly via the regulation of UCP1 protein stability and ROS levels.

## Experimental procedures

### Generation of mutant mice and sample collection

Adipocyte-specific NRDC-deficient mice were generated by crossing *Nrdc*^*flox/flox*^ mice (Accession No. CDB1019K, http://www.clst.riken.jp/arg/mutant%20mice%20list.html)^22^ with mice harboring the transgene expressing Cre recombinase under the control of mouse adiponectin promoter/enhancer regions within the BAC transgene (B6;FVB-Tg(Adipoq-cre)1Evdr/J, Adipo-Cre)^24^. Adipo-Cre mice were obtained from the Jackson Laboratory. Mice were housed in environmentally controlled rooms at the Institute of Laboratory Animals, Graduate School of Medicine, Kyoto University or at the Research Center for Animal Life Science, Shiga University of Medical Science under the Institute’s guidelines for animal and recombinant DNA experiments. Animal protocols were approved by the Animal Research Committee, Graduate School of Medicine, Kyoto University and the Animal Care and Use Committee of the Shiga University of Medical Science. Female mice were used for all analyses unless otherwise indicated. To obtain samples for the biochemical analysis, mice were deeply anesthetized by isoflurane and euthanized by cervical dislocation, and their organs, such as BAT, inguinal white adipose tissue (iWAT), hearts, and livers, were then removed and immediately frozen in liquid nitrogen. Regarding the histological analysis, mice were deeply anesthetized by isoflurane and transcardially perfused with 4% paraformaldehyde [volume %] in 0.1 M phosphate-buffered saline. The study is reported in accordance with ARRIVE guidelines (https://arriveguidelines.org).

### Telemetric body temperature monitoring and respiratory gas analysis

Telemetric body temperature monitoring was performed on conscious mice using a wireless ETA-F10 Mouse Implantable Transmitter System by Data Science International (New Brighton, MN, US) as previously described^21^ in the same chamber in which oxygen consumption (VO_2_) was measured at 23 or 30 °C. Briefly, the transmitter was implanted into the abdominal cavity of mice under anesthesia. Mice then recovered for at least 5 days before data collection. Telemetry data were continuously collected for 2 days and analyzed using HEM-Evolution software (Notocord systems) for a basal core body temperature. Twelve-week-old *Nrdc*^fl/fl^ and Adipo-KO mice were held individually in a chamber at 23 or 30 °C for 1 week in order to attain a constant respiratory exchange ratio. A gas analysis was performed using indirect calorimetry (Comprehensive Lab Animal Monitoring System: Oxymax/CLAMS, Columbus Instruments, TX, US). A β_3_-adrenergic agonist (BRL-37344) and N-acetyl cysteine (NAC) were injected intraperitoneally at 0.80 mmol/kg and 500 mg/kg, respectively, to examine the effects of these reagents, followed by the measurement of VO_2_ for another 100 min.

### Respiratory gas analysis and BAT temperature monitoring under anesthesia

Respiratory gas analysis by Oxymax/CLAMS was started immediately after the intraperitoneal injection of urethan (1.3 g/kg). After 100 min measurement of VO_2_, a small incision was made on the back and two temperature sensing probes (Physitemp) were inserted into the interscapular BAT and rectum to measure BAT and rectum temperature. The measurement of BAT and rectum temperature was started at 120 min after urethan injection.

### Blood flow measurement

Mice were anesthetized with isoflurane inhalation and blood flow in the soles of the mice was measured using a Laser Speckle Blood Flow Imager (OMEGAZONE OZ-2; OMEGAWAVE, Inc., Tokyo, Japan).

### Cold tolerance test

In the cold tolerance test, 12-week-old *Nrdc*^fl/fl^ and Adipo-KO mice were individually housed in cages at 4 °C for 5 h. Core body temperature was measured using a rectal temperature probe (Physitemp) immediately after 5 h of cold exposure at an ambient temperature of 4 °C. Mice were then killed by cervical dislocation and BAT was collected.

### Western blot analysis

The preparation of tissue or cell extracts and a Western blot analysis were performed as previously described^14,21^. Rabbit polyclonal anti-UCP1 (Sigma), anti-PGC1α (Merk-Millipore), anti-catalase (Abcam) and anti-SOD1 antibody (ProteinTech) were used as the primary antibodies. Rat monoclonal anti-NRDC antibodies (#1, #135) were raised in our laboratory^21^. Anti-α-tubulin (ProteinTech) and anti-β actin (Sigma) antibodies were used as the loading controls. To quantify the intensity of signals, a densitometric analysis was performed using ImageJ software.

### Histological procedures and immunohistochemistry

The immunohistochemical staining of mouse sections was performed as previously described^18^. An anti-UCP1 (Sigma) antibody was used as the primary antibody, the specificity of which was validated by staining BAT sections from UCP1-deficient mice (Fig. 7A). The sections of wild-type and UCP1-deficient mice were kindly provided by Dr. Yuko Okamatsu & Masayuki Saito (Hokkaido University). Images were obtained by fluorescence microscopy (BZ9000, Keyence) and confocal microscopy (SP8, Leica).

### Measurement of lipids

Lipid droplets were isolated from BAT using the Lipid Droplet Isolation Kit (Abcam) in accordance with the manufacturer’s protocol. Triglycerides and total cholesterol in isolated lipid droplets were measured using LabAssay Triglyceride and LabAssay Cholesterol (Fujifilm/Wako), respectively.

### Real time-PCR

Total RNA extraction, cDNA synthesis, and a quantitative real-time PCR analysis were performed as previously described^21^. Specific primers used for the real-time PCR analysis are shown in Supplementary Table 1.

### Cell culture and transfection

Brown preadipocytes were isolated from neonatal *Nrdc*^−/−^ mice and immortalized as previously described^21^. These cells were stably transfected with a Tet-on system vector for the doxycycline-dependent expression of NRDC to establish *Nrdc*^−/−^; Tet-on-NRDC cells. Differentiation to mature adipocytes was performed as described elsewhere^25^. Briefly, cells were grown to confluence in DMEM (4.5 g l^−1^ glucose) supplemented with 10% fetal bovine serum, antibiotics, 17 µM pantothenic acid, 33 µM biotin, 100 µM ascorbic acid, 1 µM octanoic acid, and 50 nM 3,3′,5-triiodo-l-thyronine (T3) (adipocyte culture medium). Differentiation was induced by treating confluent cells for 48 h in adipocyte culture medium supplemented with 10 mg ml^−1^ insulin and 2.5 µM dexamethasone. After a 48-h induction, the culture medium was changed to adipocyte culture medium supplemented only with 10 mg ml^−1^ insulin and cells were cultured for another 96 h. All experiments with brown adipocytes were performed following full differentiation unless otherwise indicated. In the cycloheximide chase assay, mature brown adipocytes were treated with or without cycloheximide (50 μg/ml) in combination with or without MG132 (10 μM), followed by sample collection 0, 2, and 4 h after the treatment. The transfection of plasmid DNA was performed using HilyMax (Dojindo) according to the manufacturer’s protocol.

### Statistical analysis

Data are presented as mean values ± the standard deviation (SD) or standard error (SE) as specified. Differences between treatments, groups, and genotypes were analyzed using an unpaired 2-tailed Student’s *t*-test for 2 groups or a one-way ANOVA (the post hoc Tukey–Kramer HSD test) for more than 3 groups. Mixed model repeated-measures analysis was used to analyze repeated measurements. Statistical analyses were conducted using the JMP software package (SAS institute).

## Results

### Hyperthermia in adipocyte-specific NRDC-deficient mice

**Figure 1:**
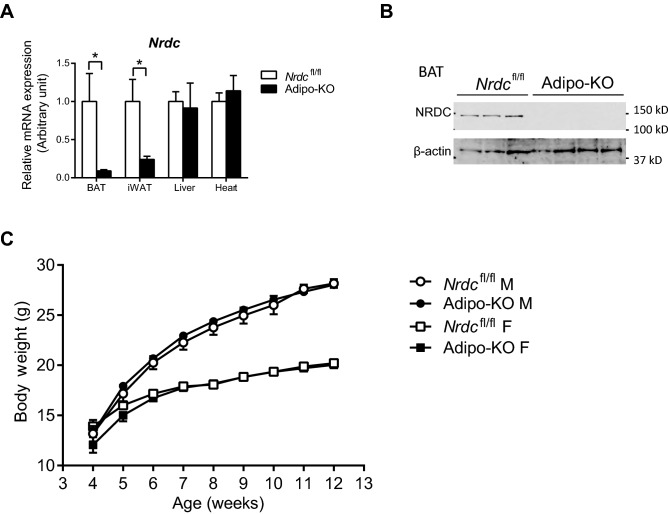
Adipocyte-specific deletion of NRDC in Adipo-KO mice. (**A**) Relative mRNA levels of NRDC in the indicated tissues from *Nrdc*^fl/fl^ and Adipo-KO mice. Results were standardized with *actb* (β-actin) mRNA levels. N = 5/genotype; postnatal day (P) 90. Data are shown as the mean + standard error (SEM), *p < 0.05. (**B**) NRDC protein levels were analyzed by the immunoblotting of BAT extracts from *Nrdc*^fl/fl^ and Adipo-KO mice. N = 3: *Nrdc*^fl/fl^, n = 4: Adipo-KO; P90. (**C**) Graph showing the body weight of *Nrdc*^fl/fl^ and Adipo-KO mice (M: male, F: female) from 4 to 12 weeks old. N = 32: *Nrdc*^fl/fl^ males, n = 34: *Nrdc*^fl/fl^ females, n = 29: Adipo-KO males, and n = 32: Adipo-KO females.

To specifically assess the role of NRDC in adipose tissue, we generated adipocyte-specific NRDC-deficient (Adipo-KO) mice by mating *Nrdc*^flox/flox^ ^22^ mice with adiponectin-Cre transgenic mice^24^. In Adipo-KO mice, NRDC mRNA levels were significantly reduced in both BAT and WAT, but not in the liver or heart (Fig. 1A). We also confirmed that the protein expression of NRDC was markedly decreased in BAT (Fig. 1B). Adipo-KO mice were born at the expected Mendelian frequency and showed no overt phenotypes. Under a normal diet, no significant differences were observed in body weight between *Nrdc*^flox/flox^ and Adipo-KO mice until the age of 12 weeks (Fig. 1C).

**Figure 2:**
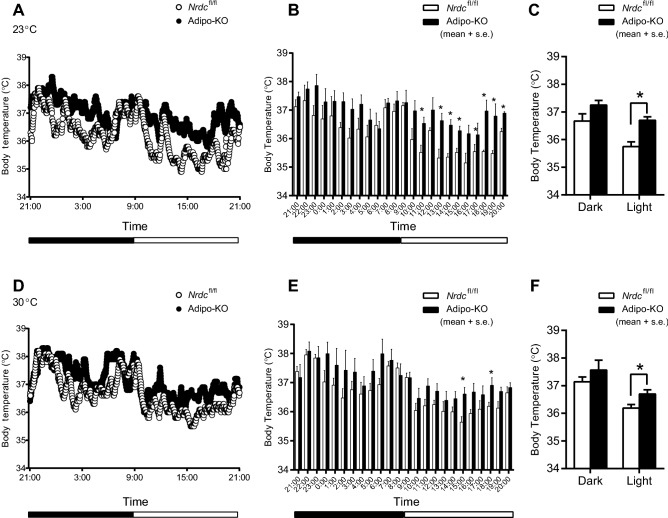
Hyperthermia in Adipo-KO mice. (**A**) Core body temperature of *Nrdc*^fl/fl^ and Adipo-KO mice at room temperature measured by the telemetry system (at 23 °C for 24 h). Dots show the average in one minute. N = 5/genotype; P90. (**B**) Hourly average of the core body temperature at room temperature (at 23 °C for 24 h). Data are shown as the mean + SEM, *p < 0.05. (**C**) Half day average of the core body temperature at room temperature (at 23 °C for 24 h). Dark; 21:00–9:00, Light; 9:00–21:00. Data are shown as the mean + SEM, *p < 0.05. (**D**) Core body temperature of *Nrdc*^fl/fl^ and Adipo-KO mice at thermoneutrality measured by the telemetry system (at 30 °C for 24 h). Dots show the average in one minute. N = 5/genotype; P90. (**E**) Hourly average of the core body temperature at thermoneutrality (at 30 °C for 24 h). Data are shown as the mean + SEM, *p < 0.05. (**F**) Half day average of the core body temperature at thermoneutrality (at 30 °C for 24 h). Dark; 21:00–9:00, Light; 9:00–21:00. Data are shown as the mean + SEM, *p < 0.05.

To characterize the thermogenic phenotype of Adipo-KO, we initially monitored their core body temperature using the telemetry system. Body temperature measured every minute at RT (23 °C) is shown in Fig. 2A, and hourly averages are described in Fig. 2B. At most of the time points indicated, Adipo-KO mice showed a significantly higher core body temperature than *Nrdc*^flox/flox^ mice, whereas the half day average was significantly higher only in the light time (Fig. 2C). We then measured body temperature at thermoneutrality (30 °C), at which mice maintain their body temperature by basal metabolism at rest and adjustments to skin blood flow^26^. Adipo-KO mice showed hyperthermia in the thermoneutral zone; however, the difference between *Nrdc*^flox/flox^ and Adipo-KO mice was smaller at 30 °C (Fig. 2D–F).

### Unchanged VO_2_, but lower physical activity in Adipo-KO mice

**Figure 3:**
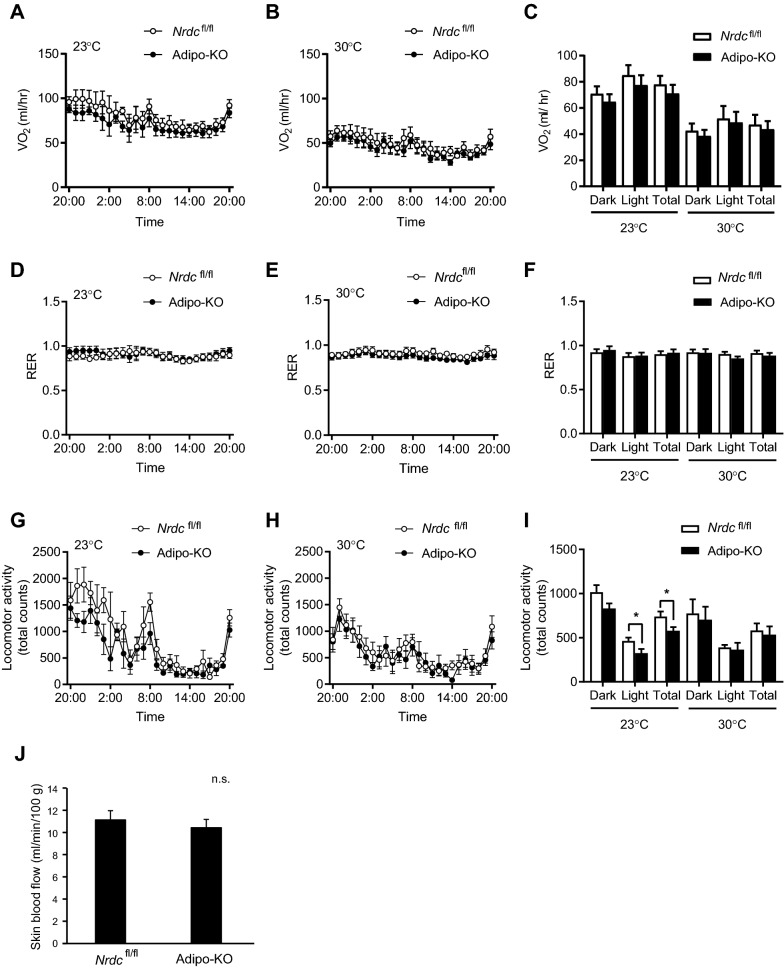
Reduced locomotor activity in Adipo-KO mice. (**A**,**B**) Hourly average of O_2_ consumption (VO_2_) of *Nrdc*^fl/fl^ and Adipo-KO mice at room temperature (**A**; 23 °C) and thermoneutrality (**B**; 30 °C). Data are shown as the mean ± standard deviation (SD). N = 6/genotype; P90. (**C**) Half day (Dark; 21:00–9:00, Light; 9:00–21:00) and full day (Total) average of VO_2_ at room temperature (23 °C) and thermoneutrality (30 °C). Data are shown as the mean ± SD. (**D**,**E**) Hourly average of the respiratory exchange rate (RER) of *Nrdc*^fl/fl^ and Adipo-KO mice at room temperature (**D**; 23 °C) and thermoneutrality (**E**; 30 °C). Data are shown as the mean ± SD. N = 6/genotype; P90. (**F**) Half day (Dark; 21:00–9:00, Light; 9:00–21:00) and full day (Total) average of RER at room temperature (23 °C) and thermoneutrality (30 °C). Data are shown as the mean ± SD. (**G**,**H**) Hourly average of locomotor activity in *Nrdc*^fl/fl^ and Adipo-KO mice at room temperature (**G**; 23 °C) and thermoneutrality (**H**; 30 °C). Data are shown as the mean ± SD. N = 6/genotype; P90. (**I**) Half day (Dark; 21:00–9:00, Light; 9:00–21:00) and full day (Total) average of locomotor activity at room temperature (23 °C) and thermoneutrality (30 °C). Data are shown as the mean ± SD, *p < 0.05. (**J**) Skin blood flow in the hindlimb of *Nrdc*^fl/fl^ and Adipo-KO mice at room temperature measured by laser speckle flowmetry. Data are shown as the mean + SD. n. s.; not significant.

Since the higher core body temperature in Adipo-KO mice suggested hypermetabolism, we measured VO_2_ using indirect calorimetry. No significant differences were observed in VO_2_ at 23 or 30 °C between *Nrdc*^flox/flox^ and Adipo-KO mice (Fig. 3A–C). Furthermore, no significant differences were noted in the respiratory exchange rate, suggesting no significant change in the energy source in the two genotypes (Fig. 3D–F). We then analyzed the physical activity of mice because major constituents of energy expenditure are adaptive thermogenesis and physical activity. The physical activity of Adipo-KO mice was significantly reduced at 23 °C, indicating that adaptive thermogenesis was enhanced in Adipo-KO mice (Fig. 3G–I).

Since body temperature is influenced by the balance between heat production and dissipation, hyperthermia may be due to better insulation. The level of insulation may be shown by the slope of increasing VO_2_ with decreasing ambient temperature. The lack of a significant difference in VO_2_ at 23 or 30 °C between *Nrdc*^flox/flox^ and Adipo-KO mice indicated no differences in insulation. We also examined skin blood flow as an indicator of heat dissipation in a hindlimb and found no significant differences between the genotypes (Fig. 3J). These results suggested that the higher core body temperature in Adipo-KO mice was due to enhanced thermogenesis.

### Increased VO_2_ and BAT temperature in Adipo-KO mice under anesthesia

**Figure 4:**
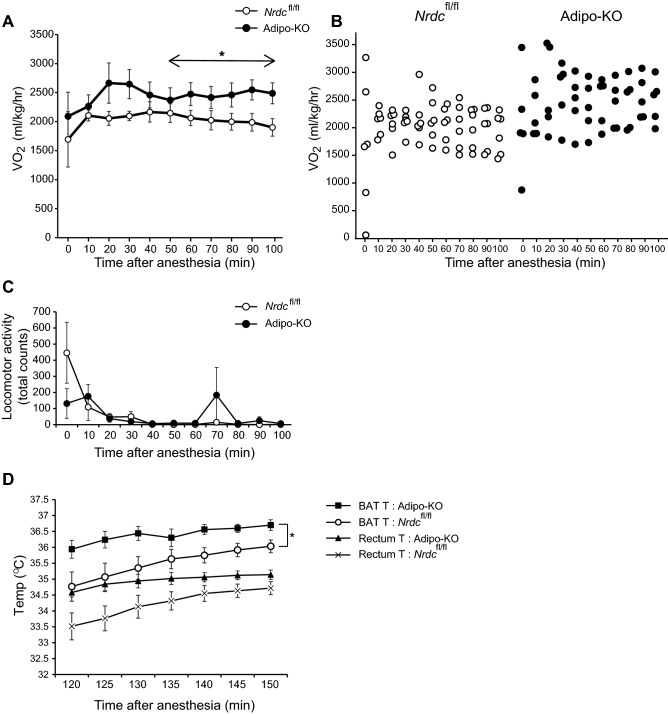
Increased VO_2_ and BAT temperature in Adipo-KO mice under anesthesia. (**A**) O_2_ consumption (VO_2_) of *Nrdc*^fl/fl^ and Adipo-KO mice under anesthesia. Data are shown as the mean ± SEM. Repeated measurements of VO_2_ were statistically analyzed by mixed models method. The model included VO_2_ as the dependent variable, with group "genotype", time, and group-by-time interaction as fixed effect, and body weight as a covariate. Mouse identification number was included as a random effect. N = 6, *Nrdc*^fl/fl^, n = 5, Adipo-KO; P90-P200, *p < 0.05. (**B**) VO_2_ in *Nrdc*^fl/fl^ and Adipo-KO mice under anesthesia. The VO_2_ values of each mouse are plotted. (**C**) Locomotor activity of *Nrdc*^fl/fl^ and Adipo-KO mice under anesthesia. Data are shown as the mean ± SEM. N = 6, *Nrdc*^fl/fl^, n = 5, Adipo-KO; P90-P200. (**D**) BAT and rectum temperature of *Nrdc*^fl/fl^ and Adipo-KO mice under anesthesia. Data are shown as the mean ± SEM. Repeated measurements of temperatures were statistically analyzed by mixed models method as described in (**A**). N = 6, *Nrdc*^fl/fl^ , n = 5, Adipo-KO; P90-P200. *p < 0.05.

To exclude the effect of physical activity on energy expenditure, we next measured VO_2_ and BAT temperature in *Nrdc*^flox/flox^ and Adipo-KO mice under anesthesia by urethan. VO_2_ in Adipo-KO mice was higher all the time after urethan injection (time 0) and there was a significant difference in the interaction between genotype and time between 50 and 100 min after the anesthesia, during which body movements ceased and the oxygen consumption values stabilized (Fig. 4A–C). After the measurement of VO_2_, we measured BAT temperature by inserting a probe with the simultaneous measurement of the core body temperature (rectum). BAT temperature was consistently higher than the rectum temperature by 1–1.5 °C, and BAT and core temperatures changed in parallel. Notably, BAT temperature in Adipo-KO mice was significantly higher than that of control mice, indicating that BAT thermogenesis is enhanced in Adipo-KO mice (Fig. 4D). Heart rate under anesthesia was measured as an index of sympathetic activity, but no significant difference was observed between *Nrdc*^flox/flox^ and Adipo-KO mice (605.8 ± 18.4/min in *Nrdc*^flox/flox^ v.s 635.4 ± 18.2/min in Adipo-KO, p = 0.29).

### Reduced mRNA expression of UCP1 in Adipo-KO BAT despite hyperthermia

**Figure 5:**
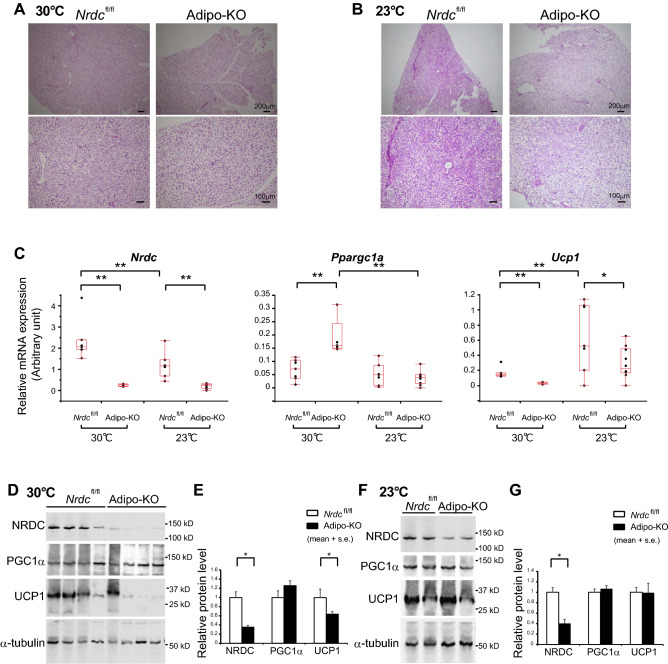
Reduced UCP1 expression in Adipo-KO BAT despite hyperthermia. (**A**,**B**) Representative images of the H-E staining of BAT sections from *Nrdc*^fl/fl^ and Adipo-KO mice at 30 °C (**A**) and 23 °C (**B**). P90: Scale bar, 100 µm (upper panels) and 200 µm (lower panels) as indicated. (**C**) Relative mRNA levels of thermogenic genes (*Nrdc*, *Ppargc1a*, and *Ucp1*) in *Nrdc*^fl/fl^ and Adipo-KO BAT at 23 or 30 °C. Results were standardized with *actb* mRNA levels (in arbitrary units), as shown in box and whisker plots. Boxes represent interquartile ranges and whiskers display the 10th and 90th percentiles. N = 7, *Nrdc*^fl/fl^ at 23 °C, n = 10, Adipo-KO at 23 °C, n = 7, *Nrdc*^fl/fl^ at 30 °C, n = 5, Adipo-KO at 30 °C. Statistical analyses were initially performed using an ANOVA followed by the Tukey HSD test. *p < 0.05, **p < 0.01. (**D**) Immunoblot analysis of total BAT extracts from *Nrdc*^fl/fl^ and Adipo-KO mice kept at 30 °C with the indicated antibodies. N = 4/genotype, P90. (**E**) Densitometric quantification of signals in the immunoblot described in (**D**). Data are shown as the mean + SEM. N = 4/genotype, P90. *p < 0.05. (**F**) Representative immunoblot analysis of total BAT extracts from *Nrdc*^fl/fl^ and Adipo-KO mice kept at 23 °C with the indicated antibodies. N = 2/genotype, P90. (**G**) Densitometric quantification of signals in the immunoblot described in (**D**). Data are shown as the mean + SEM. N = 5, *Nrdc*^fl/fl^, n = 6, Adipo-KO, P90. *p < 0.05.

To assess the thermogenic state in the BAT of Adipo-KO mice, we examined its histology and the expression levels of thermogenic genes. Despite the hyperthermic phenotype, BAT histology at thermoneutrality and RT showed no significant differences between control and Adipo-KO mice (Fig. 5A,B). Furthermore, UCP1 mRNA levels were significantly lower in Adipo-KO BAT than in control mice at 30 and 23 °C (Fig. 5C). Unexpectedly, a critical upstream regulator of UCP1 transcription, PGC1α mRNA levels at 30 °C, were significantly higher in Adipo-KO, while no significant differences were observed between *Nrdc*^flox/flox^ and Adipo-KO mice at RT (Fig. 5C). Consistent with mRNA levels, the protein expression levels of UCP1 in Adipo-KO BAT were significantly lower than those in *Nrdc*^flox/flox^ BAT at 30 °C (Fig. 5D,E). At 23 °C, which is a mildly cold environment for mice, UCP1 and PGC1α protein levels in Adipo-KO BAT were similar between control and Adipo-KO mice (Fig. 5F,G).

### The cold induction of thermogenic genes is impaired in Adipo-KO mice

**Figure 6:**
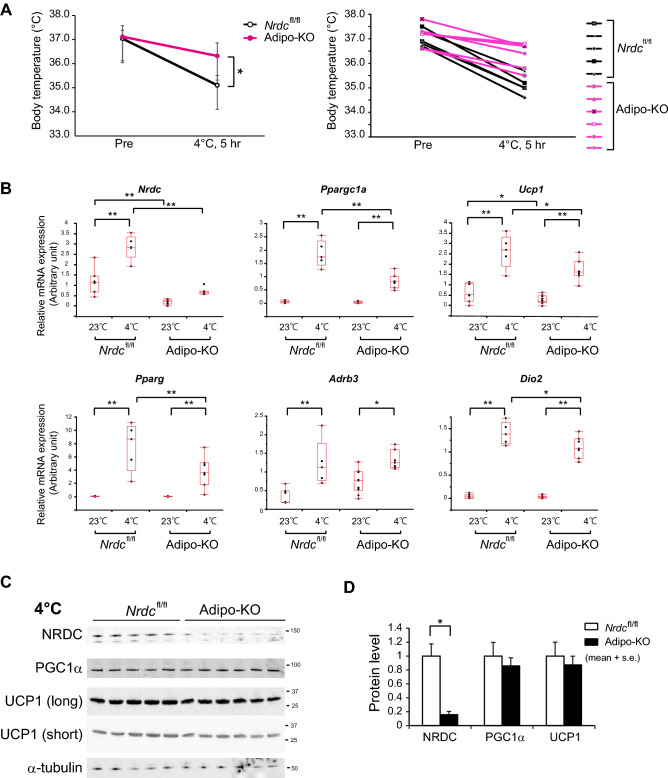
The cold induction of thermogenic genes is impaired in Adipo-KO mice. (**A**) Body temperature of 3-month-old *Nrdc*^fl/fl^ and Adipo-KO mice before and after cold (4 °C) exposure for five hours. N = 5, *Nrdc*^fl/fl^ (black line): n = 6, Adipo-KO mice (magenta line). Data represent the mean ± SD, *p = 0.0025 (right panel). Body temperature of individual mice before and after cold exposure. *Nrdc*^fl/fl^ (black line), Adipo-KO mice (magenta line) (left panel). (**B**) Relative mRNA levels of thermogenic genes (*Nrdc*, *Ppargc1a*, *Ucp1*, *Pparg*, *Adrb3*, and *Dio2*) in *Nrdc*^fl/fl^ and Adipo-KO BAT at 23 or 4 °C. Results were standardized with *actb* mRNA levels (in arbitrary units), shown in box and whisker plots, as described in Fig. 5C. N = 7, *Nrdc*^fl/fl^ at 23 °C, n = 10, Adipo-KO at 23 °C, n = 5, *Nrdc*^fl/fl^ at 4 °C, n = 7 Adipo-KO at 4 °C. Statistical analyses were initially performed by ANOVA followed by the Tukey HSD test. *p < 0.05, **p < 0.01. (**C**) Immunoblot analysis of total BAT extracts from *Nrdc*^fl/fl^ and Adipo-KO mice after cold exposure with the indicated antibodies. N = 5, *Nrdc*^fl/fl^ : n = 6, Adipo-KO mice, P90. (**D**) Densitometric quantification of signals in the immunoblot described in (**D**). Data are shown as the mean + SEM. *p < 0.05.

**Figure 7:**
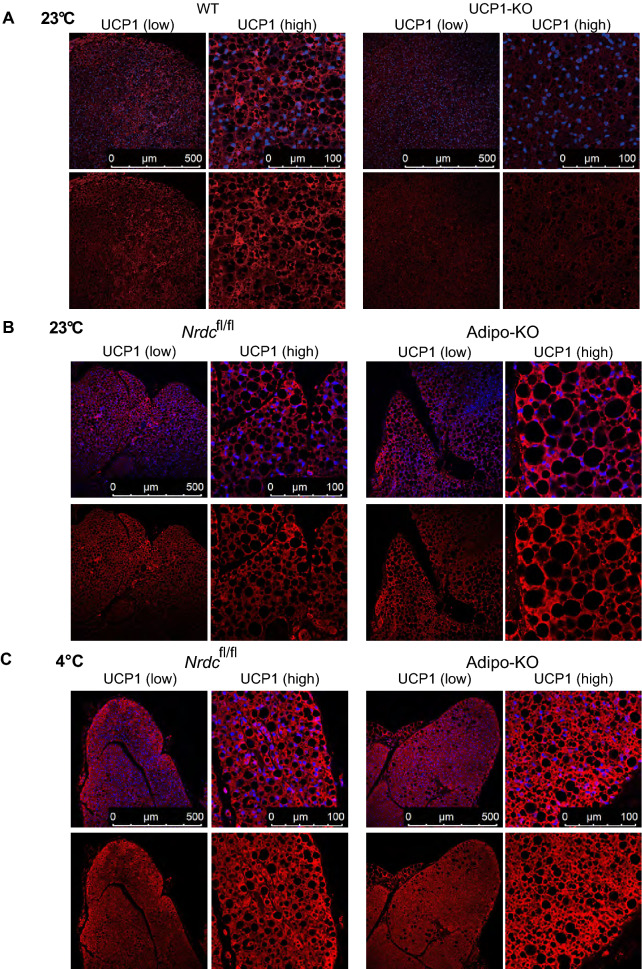
UCP1 expression in BAT at the ambient temperature 23 °C and 4 °C. (**A**) BAT sections from *wild-type* and UCP1-deficient mice were stained with the anti-UCP1 antibody (Red) and 4′,6-diamidino-2-phenylindole (DAPI; blue). P90: Scale bar, 500 µm (low magnification), 100 µm (high magnification). (**B**) BAT sections from *Nrdc*^fl/fl^ and Adipo-KO mice kept at 23 °C were stained with the anti-UCP1 antibody (Red) and 4′,6-diamidino-2-phenylindole (DAPI; blue). P90: Scale bar, 500 µm (low magnification), 100 µm (high magnification). (**C**) BAT sections from *Nrdc*^fl/fl^ and Adipo-KO mice after 5 h of cold exposure (4 °C) were stained with the anti-UCP1 antibody (Red) and 4′,6-diamidino-2-phenylindole (DAPI; blue). P90: Scale bar, 500 µm (low magnification), 100 µm (high magnification).

To clarify the role of NRDC in adipocytes on cold-induced BAT adaptive thermogenesis, we examined the cold tolerance of Adipo-KO mice at 4 °C for 5 h. The measurement of core body temperature showed that Adipo-KO mice were significantly more tolerant to the cold environment than control mice (Pre-exposure: *Nrdc*^flox/flox^ 37.04 ± 0.34 °C versus Adipo-KO 37.12 ± 0.46 °C; no significant difference, Post-exposure: 35.10 ± 0.4 °C versus 36.32 ± 0.54 °C; p = 0.0025) (Fig. 6A). In control mice, the mRNA levels of Ucp1, PGC1α, and other thermogenesis-related genes were markedly induced by cold exposure. In contrast, the cold induction of these thermogenic genes, except for the β3-adrenergic receptor, was significantly weaker in Adipo-KO mice than in *Nrdc*^flox/flox^ mice (Fig. 6B). However, UCP1 and PGC1α protein expression, measured semi-quantitatively by Western blotting, was similarly induced by cold in control and Adipo-KO mice, indicating a discrepancy in the mRNA and protein levels of UCP1 (Fig. 6C,D). UCP-1 protein expression was also evaluated by immunofluorescent staining. First, we validated the anti-UCP1 antibody for immunohistochemistry using BAT sections from UCP1-deficient mice, and found that the staining is highly specific for UCP1 (Fig. 7A). The immunohistochemical staining demonstrated an equivalent level of staining in *Nrdc*^flox/flox^ and Adipo-KO mice at 23 and 4 °C (Fig. 7B,C). We also examined lipid contents, triglycerides, and total cholesterol in the BAT extract, and found no significant differences in lipid levels between *Nrdc*^flox/flox^ and Adipo-KO BAT (Supplementary Fig. 1). Our observation of UCP1 expression at 4 and 23 °C suggests that the translated UCP1 protein is more stable in Adipo-KO mice than in control mice.

### NRDC promotes the protein degradation of UCP1

**Figure 8:**
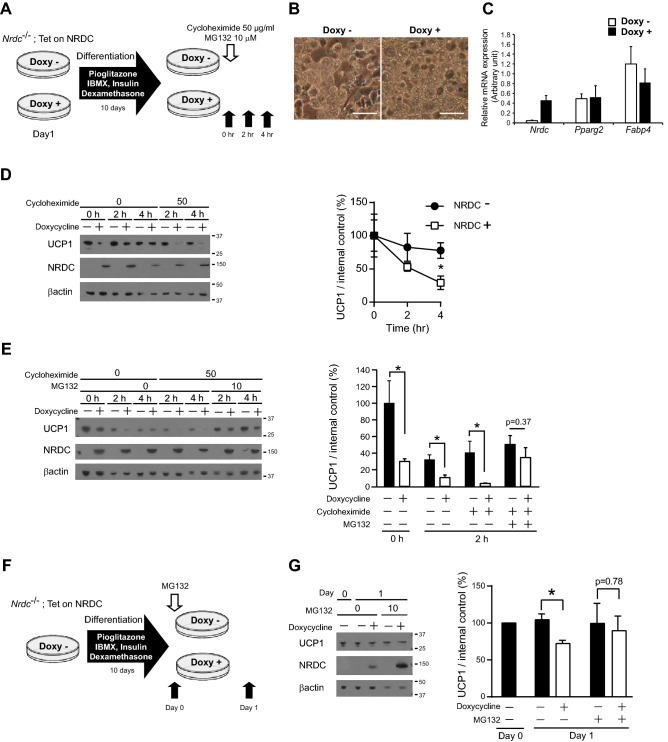
NRDC promotes the protein degradation of UCP1. (**A**) Time schedule of doxycycline (Doxy)-induced NRDC expression in *Nrdc*^−/−^; Tet-on-NRDC cells, adipocytic differentiation, and the cycloheximide chase assay. Black arrows show the time points for sample collection after the treatment with cycloheximide and MG132. (**B**) Pictures of *Nrdc*^−/−^; Tet-on-NRDC cells after adipocytic differentiation with (right) or without (left) Doxy-induced NRDC expression. Scale bar, 100 µm. (**C**) Relative mRNA levels of *Nrdc*, *Pparg2* and *Fabp4* in *Nrdc*^−/−^; Tet-on-NRDC cells after adipocytic differentiation with or without Doxy-induced NRDC expression. Results were standardized with *actb* mRNA levels (in arbitrary units) and shown as the mean + SEM. N = 5. (**D**) Representative immunoblot image of the cycloheximide chase assay (Left panel). Signals in the immunoblot are quantified by densitometry and shown as a percentage relative to the value at time point 0 (Right panel). Data are shown as the mean (%) ± SEM of 4 independent experiments, *p < 0.05. (**E**) Representative immunoblot image of the cycloheximide chase assay with or without the MG132 treatment (described in A; Left panel). Signals in the immunoblot are quantified by densitometry and shown as a percentage relative to the value at timepoint 0 (Right panel). Data are shown as the mean (%) ± SEM of 4 independent experiments, *p < 0.05. (**F**) Time schedule of the adipocytic differentiation of *Nrdc*^−/−^; Tet-on-NRDC cells and Doxy-induced NRDC expression with or without the MG132 treatment. Black arrows show the time points of sample collection. (**G**) Representative immunoblot image of the analysis described in (**F**) (Left panel). Signals in the immunoblot are quantified by densitometry and shown as a percentage relative to the value on Day 0 (Right panel). Data are shown as the mean (%) ± SEM of 3 independent experiments, *p < 0.05.

To assess whether NRDC contributes to UCP1 protein stability, we established brown adipocyte cell lines in which the expression of NRDC may be temporarily regulated. Specifically, brown preadipocytes isolated from *Nrdc*^−/−^ mice and immortalized^21^ were stably transfected with the Tet-on system vector for the doxycycline-dependent reintroduction of NRDC (*Nrdc*^−/−^; Tet-on-NRDC cells). *Nrdc*^−/−^; Tet-on-NRDC cells, treated with or without doxycycline, were fully differentiated into mature brown adipocytes and then treated with or without cycloheximide for 4 h (Fig. 8A). In this experimental setting, doxycycline / the presence or absence of NRDC did not affect the differentiation state of adipocytes, as demonstrated by the appearance (Fig. 8B) and mRNA expression of Pparg2 and Fabp4 (Fig. 8C). The cycloheximide chase assay revealed that UCP1 protein expression levels were significantly higher in NRDC-depleted cells than in doxycycline-treated NRDC-positive cells (Fig. 8D). These results suggest that NRDC promotes the protein degradation of UCP1. We then examined the effects of MG132, a potent proteasome inhibitor, on NRDC-induced UCP1 protein degradation. The simultaneous treatment of fully differentiated *Nrdc*^−/−^; Tet-on-NRDC cells with MG132 and cycloheximide clearly prevented cycloheximide-induced decreases in UCP1 expression, suggesting that NRDC promotes the degradation of UCP1 through the ubiquitin–proteasome pathway (Fig. 8E). In the next experiment, we treated cells with doxycycline after the completion of *Nrdc*^−/−^; Tet-on-NRDC cell differentiation (Fig. 8F). The 1-day induction of NRDC significantly decreased UCP1 protein expression levels, which were restored by the MG132 treatment (Fig. 8G). Collectively, these results suggest that the impaired cold induction of UCP1 mRNA was compensated by its protein stabilization in Adipo-KO BAT.

### NRDC is involved in the ROS-mediated regulation of thermogenesis

**Figure 9:**
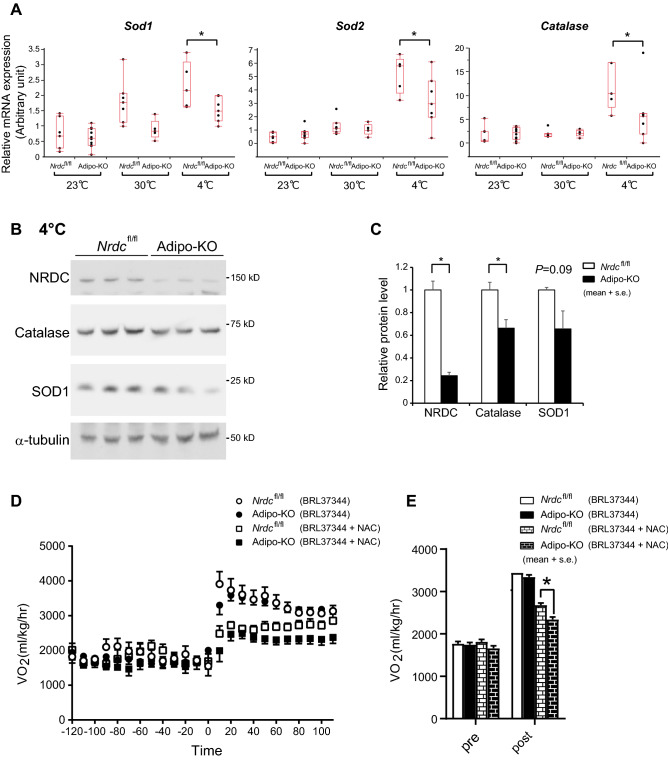
NRDC is involved in the ROS-mediated regulation of thermogenesis. (**A**) Relative mRNA levels of ROS defense genes (*sod1*, *sod2*, and *catalase*) in *Nrdc*^fl/fl^ and Adipo-KO BAT at 23, 30, and 4 °C. Results were standardized with *actb* mRNA levels (in arbitrary unit), shown in box and whisker plots as described in Fig. 5C. N = 7, *Nrdc*^fl/fl^ at 23 °C, n = 10, Adipo-KO at 23 °C, n = 7, *Nrdc*^fl/fl^ at 30 °C, n = 5, Adipo-KO at 30 °C, n = 5, *Nrdc*^fl/fl^ at 4 °C, and n = 7 Adipo-KO at 4 °C. Statistical analyses were initially performed by ANOVA followed by the Tukey HSD test. *p < 0.05. (**B**) Immunoblot analysis of total BAT extracts from *Nrdc*^fl/fl^ and Adipo-KO mice after cold exposure with the indicated antibodies. N = 3/genotype; P90. (**C**) Signals in the immunoblot (**B**) are quantified by densitometry and shown relative to the value in *Nrdc*^fl/fl^ mice. Data are shown as the mean ± SEM. *p < 0.05. (**D**) VO_2_ in *Nrdc*^fl/fl^ and Adipo-KO mice at 30 °C. At time 0, mice were peritoneally injected with a β3 agonist (BRL37344) with or without N-acetyl cysteine (500 mg/kg). Dots show the mean every 10 min ± SE. N = 6, *Nrdc*^fl/fl^ with BRL37344, n = 9, Adipo-KO with BRL37344, n = 5, *Nrdc*^fl/fl^ with BRL37344 + NAC, n = 6, Adipo-KO with BRL37344 + NAC. (**E**) Total time (2 h) average of VO_2_ before and after the treatment with BRL37344 ± NAC. Data are shown as the mean + SEM. *p < 0.05.

Cold exposure induces the generation of mitochondrial ROS, which is indispensable for UCP1-dependent thermogenesis in BAT^10^. To test whether NRDC is involved in the regulation of ROS, we analyzed the mRNA levels of ROS-related genes in BAT. Among these genes, *sod1*, *sod2*, and *catalase*, which are important for the ROS defense system, were significantly lower in Adipo-KO BAT after cold exposure (Fig. 9A). Consistently, protein expression of catalase and SOD1 showed a tendency to decrease in Adipo-KO (Fig. 9B,C). These results suggest that cold-induced ROS production was increased in Adipo-KO BAT. To assess the involvement of NRDC in ROS-mediated in vivo thermogenesis, we measured VO_2_ in *Nrdc*^flox/flox^ and Adipo-KO mice in the presence or absence of NAC, a ROS scavenger. While BRL37344, a β3 agonist, similarly increased VO_2_ in control and Adipo-KO mice, the inhibitory effects of the simultaneous treatment with NAC were significantly stronger in Adipo-KO mice (Fig. 9D,E). These results indicated that the dependence on ROS by BAT thermogenesis was greater in Adipo-KO mice than in *Nrdc*^flox/flox^ mice.

## Discussion

In the present study, we demonstrated that the core body temperature of adipocyte-specific NRDC-deficient mice was significantly higher than that of control mice. Body temperature in wild-type mice is strictly maintained by the balance between heat production and dissipation. Therefore, hyperthermia in Adipo-KO mice may be attributed to excessive heat production or reduced heat dissipation. We assessed VO_2_ as total energy expenditure and physical activity, and found that the latter was reduced in Adipo-KO mice. Since thermogenesis and physical activity are major constituents of energy expenditure, these results indicate that thermogenesis was enhanced in Adipo-KO mice. Moreover, the measurement of VO_2_ and BAT temperature under anesthesia directly indicated that BAT thermogenesis is enhanced in Adipo-KO mice. We also evaluated insulation in Adipo-KO mice by measuring VO_2_ at different ambient temperatures of 23 and 30 °C. No significant differences were observed in insulation because VO_2_ did not markedly differ at either ambient temperature between control and Adipo-KO mice. Collectively, these results suggest that hyperthermia in Adipo-KO mice was due to increased heat production.

Our previous findings revealed higher oxygen consumption in NRDC whole-body knockout (*Nrdc*^*–/–*^) mice at 23 and 30 °C, albeit the difference in energy expenditure between wild-type (*Nrdc*^+*/*+^) and *Nrdc*^*–/–*^ mice was markedly smaller at 30 °C^21^. Since the slope of increasing metabolism with decreasing ambient temperature reflects the level of insulation, these results suggest that *Nrdc*^*–/–*^ mice are less insulated than *Nrdc*^+*/*+^ mice. The present results showed no significant differences in insulation between *Nrdc*^flox/flox^ and Adipo-KO mice, indicating that NRDC in non-adipose tissue is responsible for the regulation of thermal insulation.

For the body temperature homeostasis, the central set point is another critical factor. We previously reported the lowered set point of body temperature in *Nrdc*^*–/–*^ mice. Although *Nrdc*^*–/–*^ mice showed normal thermogenic capacity at around the thermoneutrality, the body temperature stayed 1.5 °C lower than that of *Nrdc*^+*/*+^ mice at the ambient temperature 30 °C, and even at 33 °C^21^. A shift in the set point could be one reason for the higher body temperature in Adipo-KO mice. Certainly, although there should be an extra capacity for heat dissipation at 23 °C, body temperature in Adipo-KO mice was still maintained higher than the control mice, suggesting that the set point of BT is higher in Adipo-KO mice. Since NRDC deficiency in Adipo-KO mice is restricted to adipose tissue, if it affects the set point, the remote factors from adipose tissue would be important.

It currently remains unclear why the physical activity of Adipo-KO mice is reduced. Although NRDC in neuronal cells critically regulates myelination and axonal maturation^18^, the effects of the deletion of NRDC in neurons is not likely because Cre recombinase in adiponectin-Cre mice is exclusively expressed in adipose tissue^27^. While the contribution of dysregulated adipokine secretion cannot be ruled out, another possibility is that hyperthermia itself is the cause of hypokinesia. The difference in core body temperature between control and Adipo-KO mice was smaller at thermoneutrality, at which no significant difference was observed in physical activity. These results suggest the importance of the primary effect of hyperthermia on physical activity.

Adipo-KO mice showed increased cold tolerance; however, the cold induction of the mRNA levels of UCP1 and several other thermogenesis-related genes was weaker. In the present study, we identified two possible factors under NRDC-deficient conditions that induced thermogenesis despite the low mRNA levels of UCP1; the enhanced stability of the UCP1 protein and increased ROS production. While a few studies suggested that UCP1 protein stability is regulated through the ubiquitin–proteasome pathway^8^, the underlying molecular mechanisms remain unclear. The results of the cycloheximide chase assay in vitro revealed that NRDC was involved in UCP1 protein stability via the MG132-sensitive ubiquitin–proteasome pathway. Since NRDC in *Drosophila* localizes to mitochondria and functions as a co-chaperone^28^, this metalloendopeptidase in mammals may also regulate UCP1 protein quality control. In mice, UCP1 protein stabilization by the deficiency of NRDC appears to be limited to when the ambient temperature is between room temperature and low temperature. At the thermoneutrality (30 °C), both mRNA and protein levels of UCP1 are significantly lower in Adipo-KO BAT, and there is no dissociation between mRNA and protein levels. To avoid the risk of hyperthermia, UCP1 has to be rapidly downregulated when the ambient temperature rises. Therefore, we speculate that the organism may be preparing more UCP1 degradation pathways at high ambient temperatures. These pathways may compensate for the effect of NRDC deficiency and degrade UCP1 as in normal conditions.

Recent studies have highlighted the importance of ROS production in UCP1-dependent thermogenesis. ROS levels are elevated by cold exposure or a β-adrenergic stimulus, which then activate thermogenesis in mouse BAT. Furthermore, the pharmacological depletion of ROS in vivo has been shown to impair the capacity for BAT thermogenesis^10^. The present results indicated that the ROS dependency of BAT thermogenesis was greater in Adipo-KO mice. Although we found the down-regulated expression of several genes responsible for the ROS defense system in Adipo-KO mice, the molecular mechanisms by which NRDC regulates the synthesis of ROS warrant further study.

In the present study, we focused on the role of NRDC in adipocytes on UCP1-dependent thermogenesis in BAT. However, recent studies indicated that there are many alternative systems for UCP1-independent adaptive thermogenesis, such as sarco/endoplasmic reticulum Ca^2+^-ATPase-dependent and creatine metabolism-dependent pathways^9^. The involvement of NRDC in UCP1-independent thermogenesis cannot be excluded at this point. The more detailed characterization of Adipo-KO mice will deepen our understanding of the molecular system of BAT thermogenesis.

## Supplementary Information

Supplementary Information.
