# Supplementary material for: Nardilysin in adipocytes regulates UCP1 expression and body temperature homeostasis
Source: Sci Rep. 2022 Mar 2;12:3449. doi: 10.1038/s41598-022-07379-x (PMC8891301; doi:10.1038/s41598-022-07379-x)
Supplement: Supplementary file 1 — Supplementary Information. [file 41598_2022_7379_MOESM1_ESM.pdf]

Supplementary Information

Nardilysin in adipocytes regulates UCP1 expression and body temperature homeostasis

Sayaka Saijo, Mikiko Ohno, Hirotaka Iwasaki, Shintaro Matsuda, Kiyoto Nishi, Yoshinori Hiraoka, Natsuki Ide, Takeshi Kimura, & Eiichiro Nishi*

Supplementary Figures 1-2

Supplementary Table 1

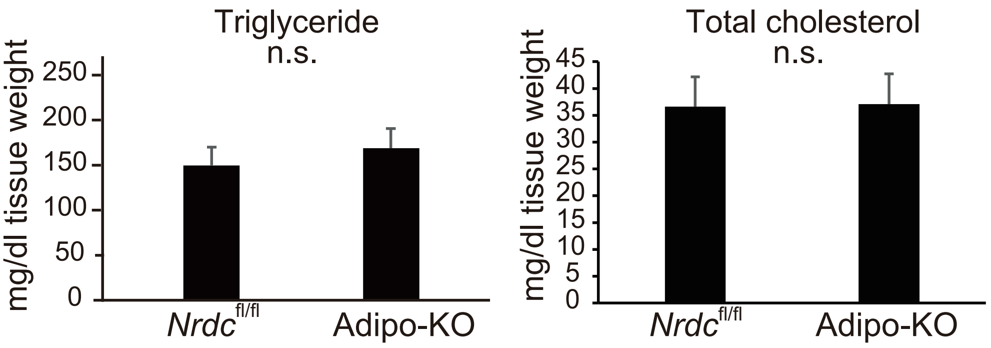

Supplementary Figure 1.

Measurement of triglyceride (left) and total cholesterol (right) levels in BAT lipid extracts from *Nrdc*$^{fl/fl}$ and Adipo-KO mice after 5 hours of cold exposure (4°C). Data are shown as the mean + SEM. N=5/genotype, P90. n.s.: Not significant.

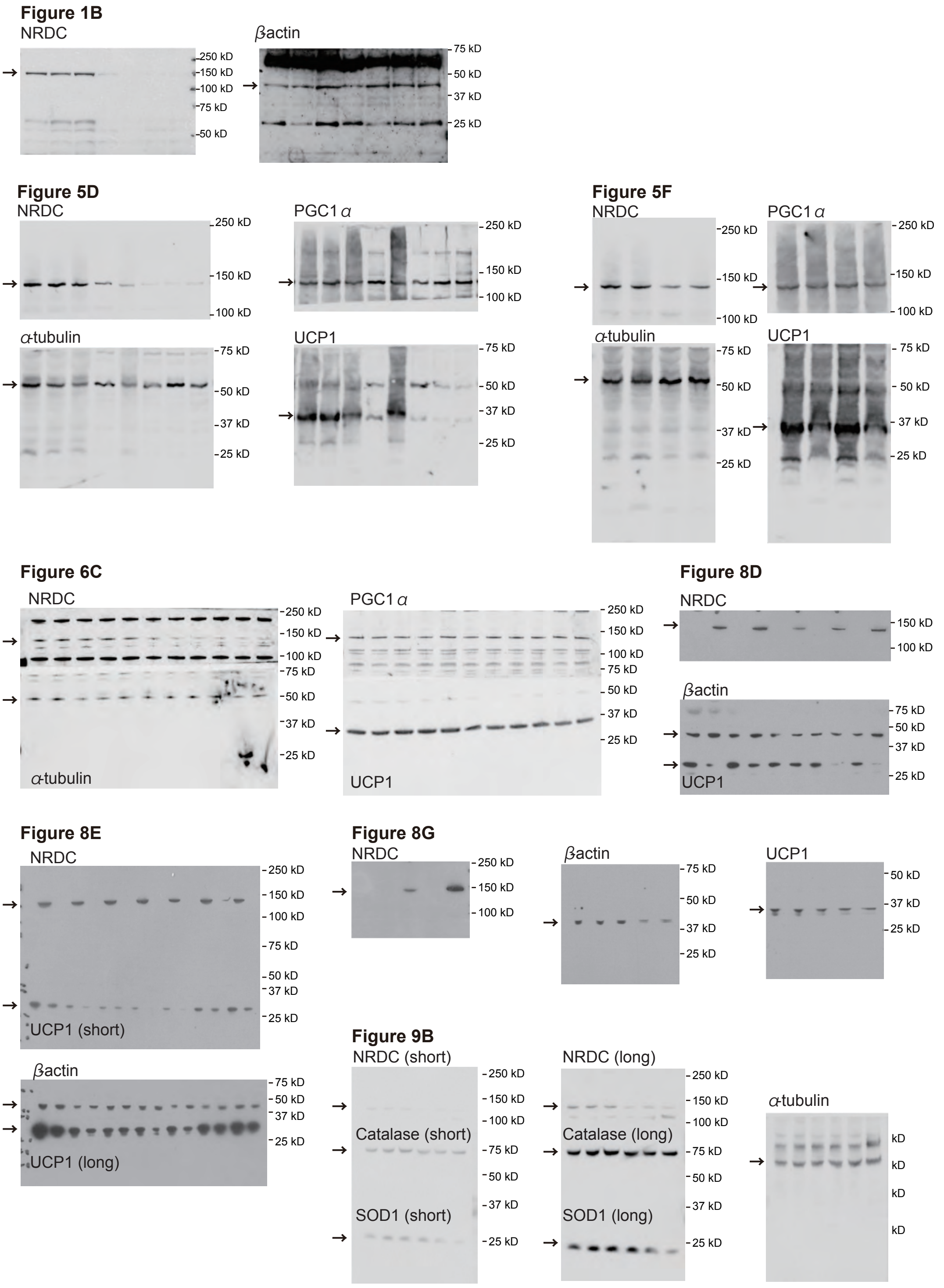

Supplementary Figure 2.

Full length blots of Figures presented in the main paper.

Supplementary Table 1. Primer sequences used for RT-PCR

Gene name	Forward (sequence 5'-3')	Reverse (sequence 5'-3')
Actb	CTGACTGACTACCTCATGAAGATCCT	CTTAATGTCACGCACGATTTCC
Nrdc	ATGGATGGCCTTTCCCTTG	CGCGAAGTTCAGCTTGTCAA
Ppargc1a	AGCCGTGACCACTGACAACGAG	GCTGCATGGTTCTGAGTGCTAAG
Ucp1	GGCATTCAGAGGCAAATCAGCT	CAATGAACACTGCCACACCTC
Pparg	GGAAGACCACTCGCATTCCTT	TCGCACTTTGGTATTCTTGGAG
Adrb3	CCACTCCGGGAACACCG	GGCAGTAGATGACCGGGTTG
Dio2	CAGTGTGGTGCACGTCTCCAATC	TGAACCAAAGTTGACCACCAG
Fabp4	AGAAGTGGGAGTGGGCTTTG	CCGCCATCTAGGGTTATGATGC
Sod1	CAAGCGGTGAACCAGTTGTG	TGAGGTCCTGCACTGGTAC
Sod2	GCCTGCACTGAAGTTCAATG	ATCTGTAAGCGACCTTGCTC
Catalase	ACCCTCTTATACCAGTTGGC	GCATGCACATGGGGCCATCA
